# Prognostic value and clinicopathological significance of serum- and tissue-based cytokeratin 18 express level in breast cancer: a meta-analysis

**DOI:** 10.1042/BSR20171145

**Published:** 2018-03-21

**Authors:** Jiangling Yang, Sicheng Gao, Jian Xu, Junfeng Zhu

**Affiliations:** 1 Department of Hepatology, Beilun Hospital of Traditional Chinese Medicine, 501 Kunlunshan Road, Beilun district, Ningbo 315800, China; 2 Department of Hepatology, Shanghai Municipal Hospital of Traditional Chinese Medicine, Shanghai University of Traditional Chinese Medicine, 274 Zhijiang Road (Middle), Shanghai 200071, China; 3 Department of Mental Diseases, Shanghai Municipal Hospital of Traditional Chinese Medicine, Shanghai University of Traditional Chinese Medicine, 274 Zhijiang Road (Middle), Shanghai 200071, China

**Keywords:** breast cancer, CK18, meta-analysis, prognosis, specimen-depended

## Abstract

Cytokeratin 18 (CK18), a type I cytokeratin of the intermediate filament family, has been associated with the prognosis of cancer patients for decades. However, its exact role in predicting the clinical outcome of breast cancer remains controversial. To comprehensively investigated the prognostic value of CK18 in breast cancer, a systematically meta-analysis was conducted to explore the association between CK18 expression and overall survival. Literature collection was conducted by retrieving electronic databases Pubmed, Cochrane Library, Web of Science, EMBASE, and OVID completely (up to January 1, 2017). Nine relevant studies with 4857 cases assessing the relationship between CK18 high expression and the outcome of breast cancer patients were enrolled in our analysis. The results indicated that the high level of CK18 expression was significantly associated with overall survival of breast cancer patients via a specimen-depended manner. Reports which used serum to detect the expression of CK18 predicted a poor outcome of breast cancer (HR = 1.24, 95%CI: 1.11–1.38, *P*<0.0001), while studies which used tissue as specimen indicated a reverse result (HR = 0.71, 95%CI: 0.60–0.84, *P*<0.00001). Moreover, overexpression of CK18 was highly relevant to advanced clinicopathological parameters of breast cancer, such as progesterone receptor, human epidermal growth factor receptor-2, tumor size, tumor stage, nodal status, and tumor grade. Taken together, the present study demonstrated that CK18 might be served as a novel biomarker to predict clinicopathological features and the outcome of breast cancer.

## Introduction

Breast cancer is the most common neoplasm damaging women’s health worldwide, which accounts for 22.9% of invasive cancers and 18.2% of all cancer deaths in women [1]. With the application of traditional biomarkers, including estrogen hormone receptors (ER) [2], progesterone receptor (PR) [3], and human epidermal growth factor receptor-2 (HER2) [4], numerous promising advances concerning the breast cancer therapy are reported. However, in order to provide maximal beneficial effects, more personalized approach with improved understanding of novel markers are encouraged. Due to the intratumoral heterogeneity of breast cancer, in combination with the mutation and evolution during the metastatic process, resistance to the molecularly therapeutic agents still remains a challenge [5,6]. Thus, it is of great importance to develop more predictive markers to establish the optimum therapeutic strategy.

It is widely accepted that cytokeratins are major structural component of epithelial cell and tissue architecture, with a highly conserved conformation during evolution [7]. The expression of cytokeratin proteins is largely determined by the epithelial cell differentiation, which could be regarded as a utility tool for distinguishing carcinomas from other types of cancer [8]. Cytokeratin 18 (CK18) belongs to a type I cytokeratin of the intermediate filament family. With its coexpressed complementary type II keratin partner, CK18 exert its function by regulating cellular processes, such as mitosis [9], apoptosis [10], and proliferation [11]. During the apoptosis, soluble CK18 (M65) and a caspase-cleaved fragment of CK18 (M30) have been released into blood [12], which could be recognized as biomarkers in the diagnosis of cancer.

CK18 has been associated with the prognosis of patients in a variety of cancers [13,14]. However, the exact predictive potential of CK18 in breast cancer still remains controversial. In one study, loss of CK18 expression was reported to be a good indicator of the poor prognosis of the breast cancer [15]. In contrast, other studies demonstrated that CK18 up-regulation was significantly associated with advanced clinical stage and poor outcome in patients with breast cancer [16,17]. Besides, the published evidence of CK18 expression for breast cancer prognosis have not been systematically reviewed. In the present study, we conducted a pooled analysis to settle these conflicting tissues, and to identify the precise role of CK18 served as a potential diagnostic biomarker.

## Material and methods

### Publication research

A systematically publication search was conducted by accessing database of Pubmed, Cochrane Library, Web of Science, EMBASE, and OVID up to January 1, 2017. Following items were used when searching the literature: (“cytokeratin 18” or “CK18” or “keratin 18” or “KRT 18 protein”) combined with (“breast cancer” or “breast carcinoma”). The references of the studies were also scanned for potential eligibility and the authors of studies were contacted if necessary. Literature search was performed by two authors (S. Gao and J. Yang) independently.

### Selection criteria

Studies were regarded as eligible when they met following criteria: (1) case–control or cohort association study; (2) focused on the relationship between cytokeratin 18 expression and prognosis of breast cancer; (3) sufficient data to extract the pooled Hazard Ratio and the corresponding confidence interval; (4) expression patterns and the detection methods were identified clearly; (5) sample size must be no less than 40. The exclusion criteria were as following: (1) insufficient data to extract; (2) case reports, reviews, letters, and expert opinions; (3) not human-based studies.

### Quality evaluation

We used the Newcastle–Ottawa scale (NOS) to assess the risk of bias and quality of the studies. The NOS tool uses nine items to evaluate every study enrolled based on three criteria: selection, comparability, and outcome. One star is awarded when they met each item and a study with final score >6 stars was considered as high quality. Two authors (J. Yang and J. Xu) performed quality assessment independently, with disagreement resolved by consensus.

### Data extraction

Interest variable data including the following information were extracted: basic characteristics (first author, publication year, country, sample size, ethnicity, follow-up time, and mean age), oncologic outcomes (survival conditions, HR estimation and corresponding 95%CI, histological stage, tumor metastasis, nodal status, ER expression, PR expression, and HER expression), and methodology (testing methods, sources of specimen and cut-off value). Software Engauge Digitizer 4.1 was used to estimate the data if a Kaplan–Meier curve was provided only. Two authors (J. Xu and J. Zhu) performed data extraction independently and discrepancies were resolved by consensus.

### Statistical analysis

The main analyses were performed by Review Manager Version 5.3 software. The hazard ratio (HR) and corresponding 95% confidence interval (CI) were pooled to estimate the association between CK18 expression and survival in breast cancer patients. The odds ratio (OR) and 95%CI were used to assess the correlation between ck18 expression and clinical parameters of patients in breast cancer. Chi-square test and *I*^2^ test were performed to measure the heterogeneity. Random-effects model or fixed-effects model was conducted according to the results of heterogeneity test. Sensitivity analysis was performed to evaluate the stability of our results. Publication bias was conducted using Begg’s and Egger’s asymmetry tests, which was performed by STATA software version 12.0.

## Results

### Selection of literature

**Figure 1:**
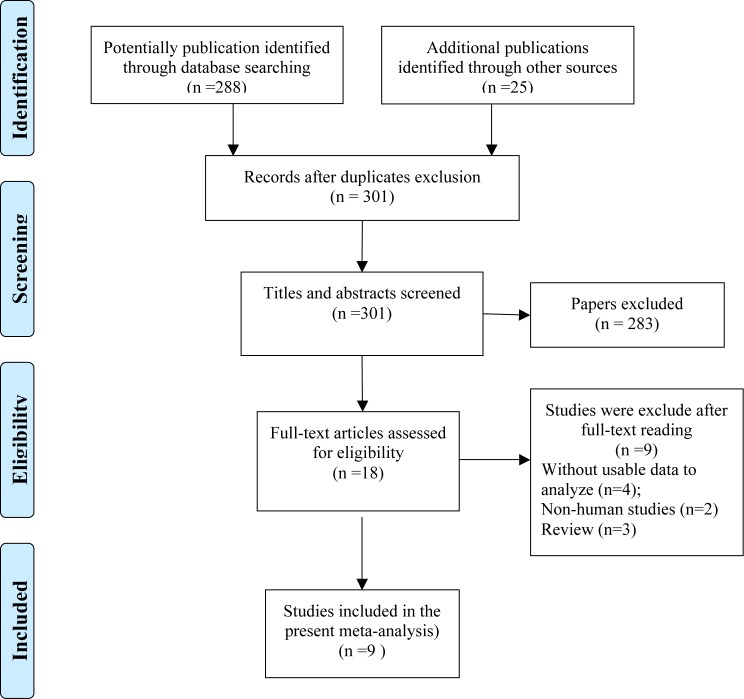
Flow diagram of the study selection process

By comprehensively publication research through database of Pubmed, Cochrane Library, Web of Science, EMBASE and OVID, a total of 301 studies were noted at first. After scanning the titles and abstracts, 283 irrelevant studies were excluded. To further evaluate the eligibility of studies, full-text reading was conducted and nine studies were removed because of the inconformity of the selection criteria. Eventually, 9 studies with 4857 cases assessing the relationship between CK18 expression and breast cancer were accepted in our analysis. A flow diagram displaying the selection process of references is shown in Figure 1.

### Basic characteristics of enrolled studies

The sample size of all accepted nine studies [18–26] ranged from 43 to 1477. Among these studies, three reports were principally originated from Germany, two from Japan, and four from China, Turkey, Sweden, and Korea respectively. Five studies used ELISA to test the expression of CK18 in breast cancer and four used IHC. Four studies were published before 2010, and the other five studies were after 2010. Of the nine studies enrolled in our meta-analysis, two of them evaluated the progress-free survival (PFS) and OS of cancer patients with CK18 aberrant expression [18,24]. Six of them only evaluated the association between elevated expression of CK18 and OS of patients [19,21–23,25,26]. One study only focused on the PFS of patients [20]. The basic characteristic of enrolled studies was listed in Table 1.

**Table 1:** Basic characteristics of the studies enrolled

No.	First author	Year	Country	Sample size	Mean Age (year)	Duration of follow-up (month)	Survival condition	Testing methods	Cut-off value	Sources	With chemotherapy	Segment type	RR (95%CI)
**1**	Shangnao Xie [18]	2014	China	975	48.5 (23–71)	NM	PFS	ELISA	80 u/l	Serum	Y	M30	1.61 (1.20–2.30)
							OS	ELISA					1.54 (1.18–2.71)
**2**	Faruk Tas [19]	2014	Turkey	80	52 (30–81)	36.5	OS	ELISA		Serum	Y	M30	1.51 (0.79–2.90)
**3**	B. K. Linderholm [20]	2013	Sweden	409	63	122.4	PFS	ELISA		Serum	Y	M30	3.30 (1.51–7.22)
**4**	Natalia Krawczyk [21]	2014	Germany	298	59 (24–90)	44 (10–88)	OS	IHC		Serum	N	M30	3.38 (1.46–7.8)
**5**	Gerhard Schaller [22]	1996	Germany	43	59 (23–91)	80 (3–98)	OS	IHC		Tissue	N	M65	0.17 (0.01–2.92)
**6**	Ute Woelfle [23]	2004	Germany	1458	NM	51 (1–150)	OS	IHC		Tissue	N	M65	0.50 (0.38–0.66)
**7**	Soo Kyung Ahn [24]	2013	Korea	1477	48 (22–89)	66.3 (0–128)	PFS	ELISA	80u/l	Serum	N	M30	1.64 (1.20–2.21)
							OS	ELISA					1.57 (1.06–2.35)
**8**	Masahiro Takada [25]	2004	Japan	72	51 (28–74)	32.4	OS	IHC		Tissue	Y	M30	0.30 (0.11–0.85)
**9**	Maria Hagg Olofssion [26]	2007	Japan	45	NM	NM	OS	ELISA		Serum	Y	M65	0.21 (0.03–1.74)

Abbreviations: ELISA, enzyme-linked immune sorbent assay; IHC, immunohistochemistry; NM, not mentioned; OS, overall survival; PFS, progress-free survival.

### Quality judgment of studies

Based on the Newcastle–Ottawa scale, the quality of included studies was assessed and a study scored six or more could be regarded as high-quality. In our meta-analysis, the scores of nine studies ranged from 6 to 9, which declared that all studies included in our meta-analysis were in compliance with high quality (Table 2).

**Table 2:** Quality assessment of studies enrolled using the Newcastle–Ottawa quality assessment scale

Study [author (year)]	Selection				Comparability	Outcome			Scores
	1	2	3	4		1	2	3	
Shangnao Xie (2014) [18]	★	★	★	–	★★	–	★	★	7
Faruk Tas (2014) [19]	★	★	★	–	★	★	★	★	7
B. K. Linderholm (2013) [20]	–-	★	★	★	★★	★	★	★	8
Natalia Krawczyk (2014) [21]	★	★	★	–	★★	–	–	★	6
Gerhard Schaller (1996) [22]	★	★	★	★	★	★	–	★	8
Ute Woelfle (2004) [23]	–	★	★	★	★★	★	★	★	8
Soo Kyung Ahn (2013) [24]	★	★	★	★	★★	★	★	★	9
Masahiro Takada (2004) [25]	★	★	★	–	★★	★	★	★	8
Maria Hagg Olofssion (2007) [26]	–	★	★	★	★★	–	★	★	7

★ Study basically meets the criteria; ★★ Study meets the criteria strongly.

### Association between CK18 overexpression and overall survival of breast cancer

**Figure 2:**
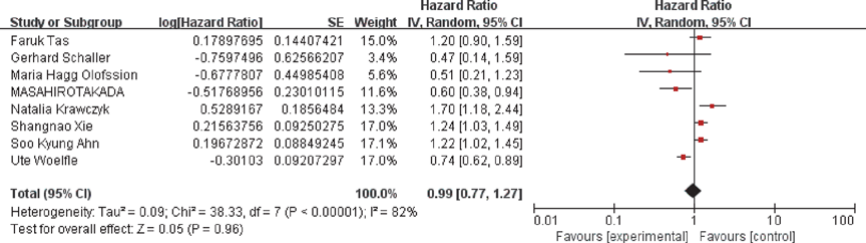
Association between high CK18 expression and the overall survival of breast cancer patients

A random-effects model was employed to conduct the analysis because of the existence of heterogeneity (*I*^2^ = 82%, *P*<0.00001). When we focused on the association between CK18 overexpression and OS of cancer patients, only eight studies were analyzed. The pooled HR was 0.99 with 95%CI: 0.77–1.27 (*P*=0.96) (Figure 2), indicating that no significant relationship was found between CK18 overexpression and OS of breast cancer. Then we conducted subgroup analysis stratified by ethnicity (Supplementary Figure S3A), median follow-up time (Supplementary Figure S3B), and mean age (Supplementary Figure S3C) with no significant correlation was found. Detailed data of overall and subgroup meta-analysis were summarized in Table 4.

### CK18 predicts overall survival of breast cancer via a specimen-depended manner

**Figure 3:**
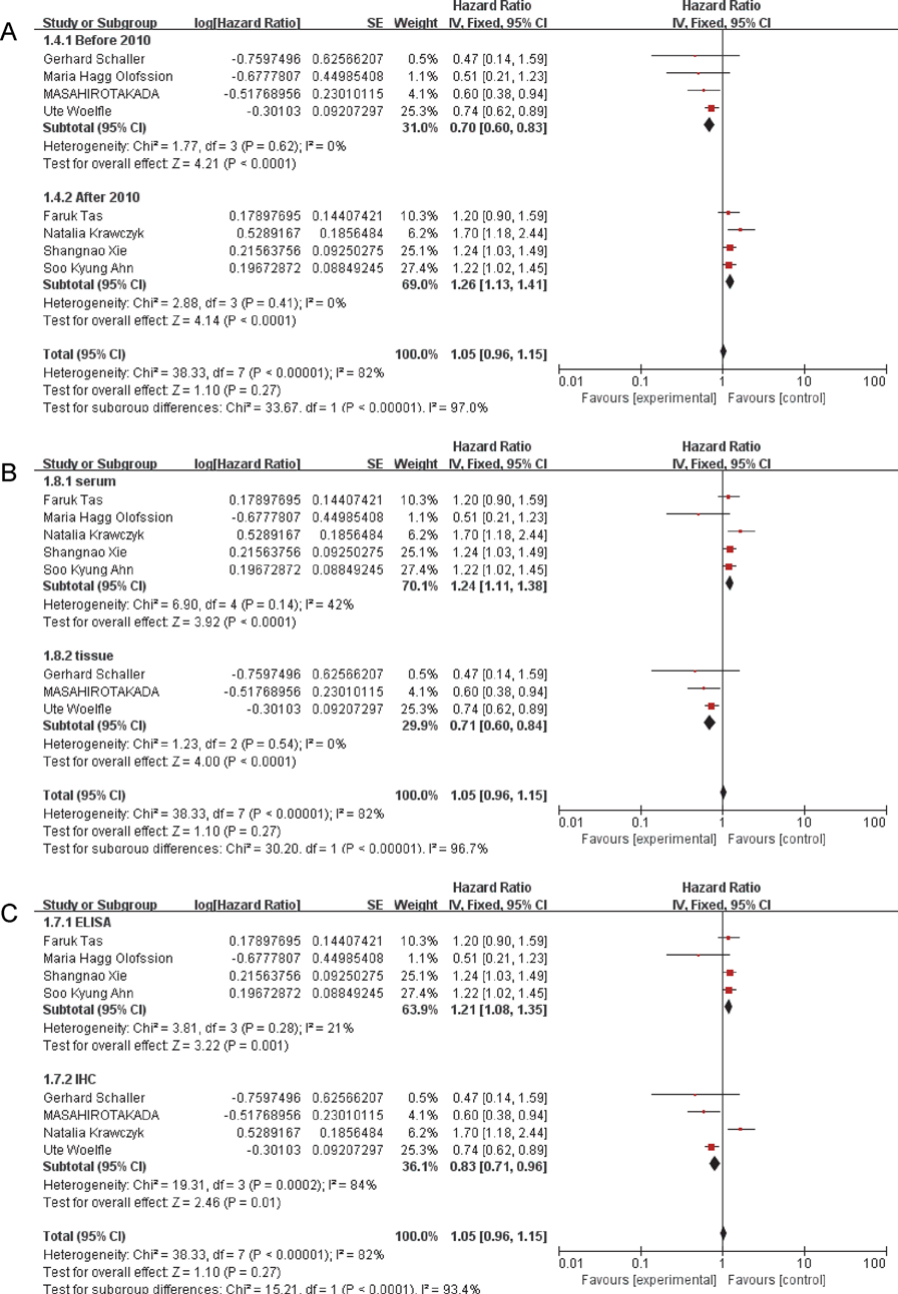
Subgroup analysis results of CK18 overexpression and breast cancer prognosis (**A**) Subgroup analysis results based on publication year. (**B**) Subgroup analysis results based on specimen sources. (**C**) Subgroup analysis results based on testing methods.

**Figure 4:**
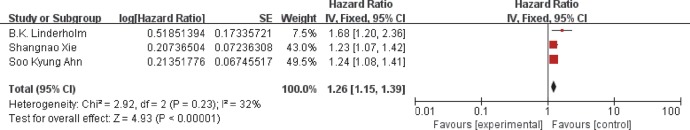
Association between CK18 overexpression and progress-free survival of breast cancer patients

As showed in Figure 3, subgroup analysis was also conducted by the following stratification: publication year, specimen sources, and testing methods. When stratified by specimen sources, the results (Figure 3B) indicated that reports which used serum to detect the expression of CK18 predicted a poor outcome of breast cancer (HR = 1.24, 95%CI: 1.11–1.38, *P*<0.0001) . However, studies which used tissue as specimen indicated a reverse result. The pooled HR (0.71) with corresponding 95%CI (0.60–0.84, *P*<0.00001 = indicated that CK18 overexpression was significantly relevant with a favorable prognosis of breast cancer patients. As for the testing methods (Figure 3C), four studies enrolled 2575 patients used ELISA to detect CK18 expression, which predicted a worse outcome of breast cancer patients (HR = 1.21, 95%CI: 1.08–1.35, *P*=0.0001) than studies used IHC (HR = 0.83, 95%CI: 0.71–0.96, *P*<0.0001). Furthermore, four studies (Figure 3A) published before 2010 indicated a good prognosis (HR = 0.70, 95%CI: 0.60–0.83, *P*<0.0001) while the studies published after 2010 showed contrary results (HR = 1.26, 95%CI: 1.13–1.41, *P*<0.00001). Besides, three studies enrolled 2861 patients predicted that CK18 overexpression was significantly associated with the PFS of breast cancer patients (HR = 1.26, 95%CI:1.15–1.39, *P*<0.00001) (Figure 4).

Moreover, keratin 18 protein in the serum has different forms that are recognized by different monoclonal antibodies. M30 detects the apoptotic fragment of krt18 but M65 detects both necrosis and apoptotic krt18 as well as full length krt18 protein. Thus, we conducted subgroup analysis based on the segment types of Keratin 18 (M30 and M65) both in serum and on tissue sections. In serum, the results (Supplementary Figure S5B) indicated that reports used M30 antibody to detect the expression of CK18 predicted a poor outcome of breast cancer (HR = 1.26, 95%CI: 1.13–1.41, *P*<0.001), which is consistent with the subgroup analysis based on specimen sources. However, we found that the studies detected serum level of Keratin 18 mostly used M30 antibody. Similarly, in tissue section, the results (Supplementary Figure S5C) indicated that reports used M30 antibody or M65 antibody both indicated a favorable outcome of breast cancer (HR_M30_ = 0.60, 95%CI: 0.38–0.94, *P*=0.02; HR_M65_ = 0.73, 95%CI: 0.61–0.87, *P*=0.0004), which is also consistent with the subgroup analysis based on specimen sources. No matter which kind of antibody used in tissue section, the results were similarly. Furthermore, we conducted a subgroup analysis according to segment types of CK18 in all enrolled studies. The results (Supplementary Figure S5A) of M30 subgroup showed that no significant relationship was found between CK18 high expression and OS of breast cancer (HR = 1.17, 95%CI: 0.95–1.45, *P*=0.13). Above all, we believed that no difference based on different monoclonal antibodies either in serum or on tissue sections.

Besides, as we all know, the CK18 level in chemotherapies of breast cancer is quite different from patients without chemotherapy and the preoperative level of CK 18 and the postoperative CK18 level should be discussed separately. However, the related information we could extracted from enrolled studies was limited. Hence, we divided the enrolled studies into two parts simply, subgroup with chemotherapy and subgroup without chemotherapy, and conducted the subgroup analysis. The results (Supplementary Figure S5D) indicated that no significant relationship was found between CK18 overexpression and outcome of cancer patients in both subgroup with chemotherapy (HR = 0.93, 95%CI: 0.65–1.33, *P*=0.70) and subgroup without chemotherapy (HR = 1.05, 95%CI: 0.69–1.59, *P*=0.84). The chemotherapy could influence the expression of CK18, but the CK18 level could not predicted the outcome of cancer patients when stratified on chemotherapy, which might be caused by the limited research. Thus, more researches were encouraged to evaluate the prognostic value of CK18.

### Correlations between CK18 high expression and clinicopathologic parameters

The detailed information evaluating the correlations between CK18 high expression and clinicopathological parameters are summarized in Table 3. According to our analysis (Supplementary Figure S1), significant relationship was found between CK18 high expression and PR (OR = 1.27, 95%CI: 1.07–1.52, *P*=0.008) (Supplementary Figure S1C), and HER2 (OR = 1.29, 95%CI: 1.01–1.64, *P*=0.04) (Supplementary Figure S1D). However, no significant relationship was observed when assessing the relationship between CK18 and ER (OR = 1.13, 95%CI: 0.93–1.36, *P*=0.22) (Supplementary Figure S1B). In order to further evaluate the relationship between CK18 and clinicopathological parameters of breast cancer, the effects of tumor stage, nodal status, and tumor grade were also pooled. Statistical analysis indicated that CK18 overexpression was highly relevant with tumor stage (OR = 2.83, 95%CI: 1.32–6.06, *P*=0.007) (Supplementary Figure S2A), nodal status (OR = 2.11, 95%CI: 1.28–3.46, *P*=0.003) (Supplementary Figure S2B), and tumor grade (OR = 1.82, 95%CI: 1.46–2.27, *P*<0.00001) (Supplementary Figure S2C). Besides, in the pooled analysis focused on tumor size, the results showed that expression of CK18 was highly different between tumor size <2 and ≥2 cm (OR = 1.37, 95%CI: 1.19–1.58, *P*<0.0001) (Supplementary Figure S2D). Three studies were pooled to investigate the relationship between age and the poor risk of differentiation and a significant association was found (OR = 1.90, 95%CI: 1.03–3.51, *P*<0.0001) (Supplementary Figure S1A).

**Table 3:** Summarized data assessing the relationship between CK18 and clinicopathological features

Categories	OR (95%CI)	*P* value	*I*^2^ (%)
Age (≥50/<50)	1.90 (1.03–3.51)	<0.0001	91
ER (Positive/Negative)	1.13 (0.93–1.36)	0.22	0
PR (Positive/Negative)	1.27 (1.07–1.52)	0.008	21
HER (Positive/Negative)	1.29 (1.01–1.64)	0.04	39
Tumor size (≥2 cm/<2 cm)	1.37 (1.19–1.58)	<0.0001	63
Tumor stage (T3,T4/T1,T2)	2.83 (1.32–6.06)	0.007	95
Nodal status (Positive/Negative)	2.11 (1.28–3.46)	0.003	87
Tumor grade (grade 3/grade 1,2)	1.82 (1.46–2.27)	<0.00001	76

Abbreviations: ER, estrogen hormone receptors; HER, epidermal growth factor receptor; PR, progesterone receptor.

**Table 4:** A summary of overall and subgroup analysis evaluating the relationship between CK18 expression and the outcome of breast cancer patients

Categories	Cohorts (*n*)	HR (95%CI)	*P* value	*I*^2^ (%)	Model types
**OS**	8 (4448)	0.99 (0.77–1.27)	0.96	82	Random-effects
**PFS**	3 (2861)	1.26 (1.15–1.39)	<0.00001	32	Fixed-effects
**Ethnicity**					Random-effects
Asian	4 (1879)	0.97 (0.72–1.32)	0.85	76	
Caucasian	4 (2569)	1.03 (0.65–1.63)	0.90	86	
**Median follow-up time (months)**					Random-effects
>60	4 (2540)	1.12 (0.89–1.42)	0.11	51	
<60	4 (1908)	0.98 (0.64–1.48)	0.91	87	
**Mean age (years)**					Random-effects
>50	5 (2497)	0.92 (0.62–1.37)	0.69	84	
<50	3 (1951)	1.18 (0.96–1.44)	0.11	47	
**Publication year**					Fixed-effects
Before 2010	4 (2780)	0.70 (0.60–0.83)	<0.0001	0	
After 2010	4 (1668)	1.26 (1.13–1.41)	<0.00001	0	
**Specimen sources**					Fixed-effects
Serum	5 (2875)	1.24 (1.11–1.38)	<0.0001	42	
Tissue	3 (1573)	0.71 (0.60–0.84)	<0.00001	0	
**Testing methods**					Fixed-effects
ELISA	4 (2575)	1.21 (1.08–1.35)	0.0001	21	
IHC	4 (1873)	0.83 (0.71–0.96)	<0.0001	84	
**Chemotherapy**					Random-effects
With	4 (1172)	0.93 (0.65–1.33)	0.70	75	
Without	4 (3276)	1.05 (0.69–1.59)	0.84	88	
**Segment type**					Random-effects
M30	5 (2902)	1.17 (0.95–1.45)	0.13	69	
M65	3 (1546)	0.72 (0.61–0.86)	0.0003	0	

### Publication bias and sensitivity analysis

**Figure 5:**
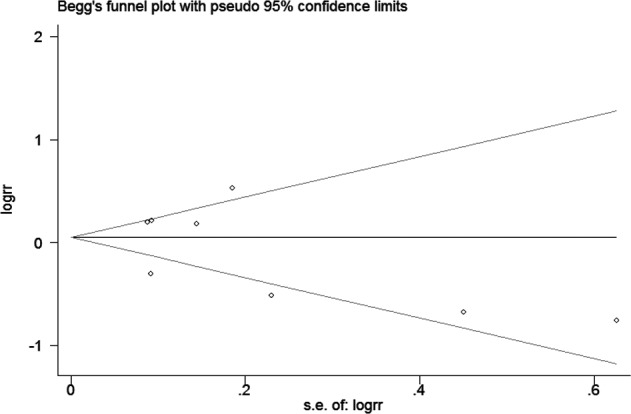
Funnel plot analysis investigating the publication bias between CK18 overexpression and cancer prognosis

**Figure 6:**
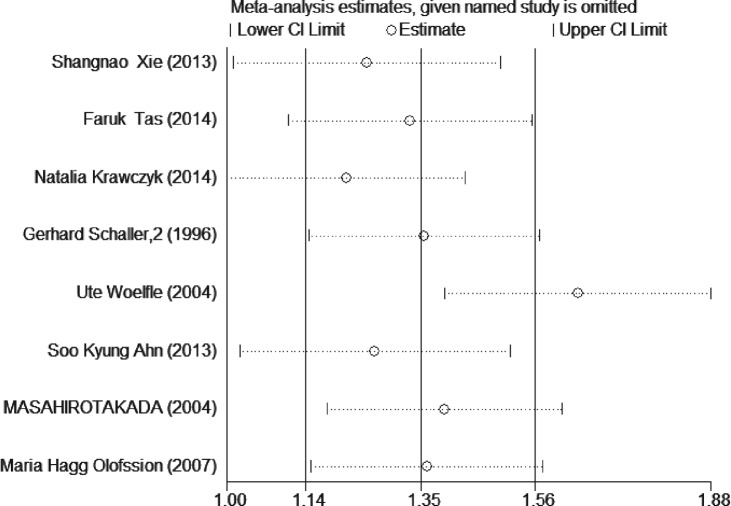
Effects of each study enrolled on pooled HRs for CK18 high expression and OS in breast cancer

The publication bias was assessed by funnel plot and Begg’s and Egger’s test. Funnel plot (Figure 5) showed no obvious asymmetry existing in our meta-analysis, which was further demonstrated by Begg’s test (*z*=0.12, *P*=0.902) and Egger’s test (*t*=0.83, *P*=0.439) (Supplementary Figure S4). In order to evaluate the stability of our results, we performed sensitivity analysis by removing a study each time. As the results indicated in Figure 6, no apparent changes were observed and our results were relatively robust.

## Discussion

As the traditional biomarkers, RP, PR, and HER2 have been used to select endocrine-sensitive breast cancers and identify breast cancer patients with metastatic disease for decades [27,28]. However, due to the heterogeneity of the breast cancer, more precise biomarkers are encouraged for the individualized treatment of recurrence and metastasis. Recently, CK18 has been associated with a significantly prognosis in breast cancers in numerous studies [29,30]. Thus, we conducted this meta-analysis to comprehensively illustrate the exact role of CK18 for predicting its prognosis value. This might be the first meta-analysis to systematically evaluate the association between CK18 and the prognosis and clinicopathological features of breast cancer.

First, we evaluate the relationship between CK18 overexpression and expression of ER, PR, and HER2 in breast cancer. Our results indicated that high expression of CK18 was significantly related with positive expression of PR and HER2, which could identify the metastatic progression of breast cancer. However, no significant relationship was found between high expression of CK18 and the positive expression of ER. In order to further investigate the clinical role of CK18, we assessed other clinicopathological features, such as tumor size (≥2 cm/ <2 cm), tumor stage (T3, T4/T1, and T2), nodal status (positive/negative), tumor grade (grade 3/ grade 1,2), and age (≥50/<50). The data showed that higher CK18 expression was positively associated with larger tumor size (≥2 cm), older age (≥50), and advance tumor metastasis of breast cancer.

A random-effects model was used to evaluate the association between CK18 overexpression and overall survival of breast cancer. HRs pooled from eight enrolled studies indicated no significant relationship between high CK18 expression and overall survival. However, due to the high heterogeneity existing in pooled effects, we conducted subgroup analysis to explore the sources. Intriguing, we found a significant relationship between CK18 high expression with the survival of breast cancer patients when stratified by publication year, testing methods, and specimen sources. According to our results, five studies used serum of patients as specimen to detect the expression of CK18, which showed a significant correlation between high CK18 expression and worse prognosis of breast cancer patients. While the other three studies which used tissue of patients to detect the expression of CK18 revealed diverse relationship. Consistently, most studies [22,23,25] published before 2010 tended to use IHC to evaluate the CK18 expression, thus leading to the diverse results when stratified by testing methods and publication year. Thus, we propose a hypothesis that CK18 overexpression was significantly associated with overall survival of breast cancer patients via a specimen-depended manner. High CK18 expression in serum was remarkable relevant with poor outcome of breast cancer patients, while when high expressed in tissue, elevated level of CK18 was significantly associated with favorable prognosis of breast cancer patients.

Similarly to our research, a meta-analysis [31] focused on the clinical values of serum CK18 in hepatitis indicated the levels of serum CK18 were elevated in hepatitis patient compared with normal controls. Another study [32] described different expression patterns of CK18 in breast tumors and investigated the possible diagnostic value of these patterns in breast cancer patients. They found a same phenomenon that a particular expression pattern of CK18 existed. The large sized tumor cells from proliferation front hand a cytoplasmic heterogeneous positive pattern of CK18, while the intensity of the tissue staining was relatively low. We thought this might because of the enormous heterogeneity of neoplasm. On the other hand, serum level of cytokeratin 18 reflects the released CK18 by dying cancer cells, while the tissue expression level of CK18 usually reflects the differentiation status of the tissues. This might explain why CK18 in serum is positively correlated with cancer progression while tissue CK18 is negatively correlated with cancer progression. The heterogeneity of neoplasm may result in the alteration of gene in peripheral blood. In other words, our results were relatively robust based on the systematically analysis and our hypothesis could be a bold and innovative conjecture. Further studies will be encouraged to confirm our hypothesis and explore the underlying mechanism.

Although a comprehensive analysis was conducted in the present study, there are still some limitations exist. First, when Engage Digitizer 4.1 was used to estimate the data, calculation errors may be unavoidable. Second, because of the testing methods, the cut-off value of CK18 expression might differ in these studies, which may cause potential bias. Third, the literatures were published openly in English, which might exclude potential research published in other languages. Fourth, because the selected reagent manufacturers, concentration of antibody and standard were inconsistent in the enrolled studies, it might also increase the publication bias. Finally, studies enrolled in our analysis were limited and corresponding sample size was relatively small. Thus, a more comprehensively analysis with large sample size was still needed to facilitate our hypothesis.

## Conclusion

Taken together, the present study indicated that overexpression of CK18 was highly correlated with advance clinicopathological parameters. Besides, the high level of CK18 expression was significantly associated with overall survival of breast cancer patients via a specimen-depended manner. CK18 might be used as a novel biomarker to predict the outcome of breast cancer. More research is encouraged to explore the underlying mechanism focused on the predictive value of CK18.

## Supporting information

**Figure S1:** Forest plot assessing the relationship between CK18 expression and clinicopathological features. (A) Association between CK18 expression and age. (B) Association between CK18 expression and ER expression. (C) Association between CK18 expression and PR expression. (D) Association between CK18 expression and HER expression.

**Figure S2:** Forest plot assessing the relationship between CK18 expression and clinicopathological features. (A) Association between CK18 expression and tumor stage. (B) Association between CK18 expression and nodal status. (C) Association between CK18 expression and tumor grade. (D) Association between CK18 expression and tumor size.

**Figure S3:** Results of subgroup analysis investigating the relationship between CK18 and overall survival of breast cancer patients. (A) Subgroup analysis based on ethnicity. (B) Subgroup analysis based on median follow-up time. (C) Subgroup analysis based on mean age.

**Figure S4:** Results of Begg’s and Egger’s test exploring the publication bias between high CK18 expression and breast cancer prognosis.

**Figure S5:** Results of subgroup analysis investigating the relationship between CK18 and overall survival of breast cancer patients. (A) Subgroup analysis based on segment types. (B) Subgroup analysis based on segment types in studies used serum to detect CK18. (C) Subgroup analysis based on segment types in studies used tissue section to detect CK18. (D) Subgroup analysis based on chemotherapy.

## Abbreviations

CK18: cytokeratin 18
ER: estrogen hormone receptor
HER2: human epidermal growth factor receptor-2
NOS: Newcastle–Ottawa scale
PR: progesterone receptor
