# Supplementary material for: Prognostic value and clinicopathological significance of serum- and tissue-based cytokeratin 18 express level in breast cancer: a meta-analysis
Source: Biosci Rep. 2018 Mar 21;38(2):BSR20171145. doi: 10.1042/BSR20171145 (PMC5861326; doi:10.1042/BSR20171145)
Supplement: Supplementary file 1 [file bsr20171145_Supp1.pdf]

Figure S1 Forest plot assessing the relationship between CK18 expression and clinicopathological features. (A) Association between CK18 expression and age. (B) Association between CK18 expression and ER expression. (C) Association between CK18 expression and PR expression. (D) Association between CK18 expression and HER expression.

Figure S2 Forest plot assessing the relationship between CK18 expression and clinicopathological features. (A) Association between CK18 expression and tumor stage. (B) Association between CK18 expression and nodal status. (C) Association between CK18 expression and tumor grade. (D) Association between CK18 expression and tumor size.

Figure S3 Results of subgroup analysis investigating the relationship between CK18 and overall survival of breast cancer patients. (A) Subgroup analysis based on ethnicity. (B) Subgroup analysis based on median follow-up time. (C) Subgroup analysis based on mean age.

Figure S4 Results of Begg's and Egger's test exploring the publication bias between high CK18 expression and breast cancer prognosis.

Figure S5 Results of subgroup analysis investigating the relationship between CK18 and overall survival of breast cancer patients. (A) Subgroup analysis based on segment types. (B) Subgroup analysis based on segment types in studies used serum to detect CK18. (C) Subgroup analysis based on segment types in studies used tissue section to detect CK18. (D) Subgroup analysis based on chemotherapy.

A

Study or Subgroup	Experimental Events	Experimental Total	Control Events	Control Total	Weight	Odds Ratio M-H, Random, 95% CI
Shangnao Xie	144	185	423	790	32.4%	3.05 [2.10, 4.43]
Soo Kyung Ahn	220	290	697	1187	34.0%	2.21 [1.65, 2.96]
Ute Woelfle	249	314	688	873	33.6%	1.03 [0.75, 1.42]
**Total (95% CI)**		**789**		**2850**	**100.0%**	**1.90 [1.03, 3.51]**
Total events	613		1808			

Heterogeneity: Tau² = 0.27; Chi² = 21.35, df = 2 (P < 0.0001); I² = 91%
Test for overall effect: Z = 2.04 (P = 0.04)

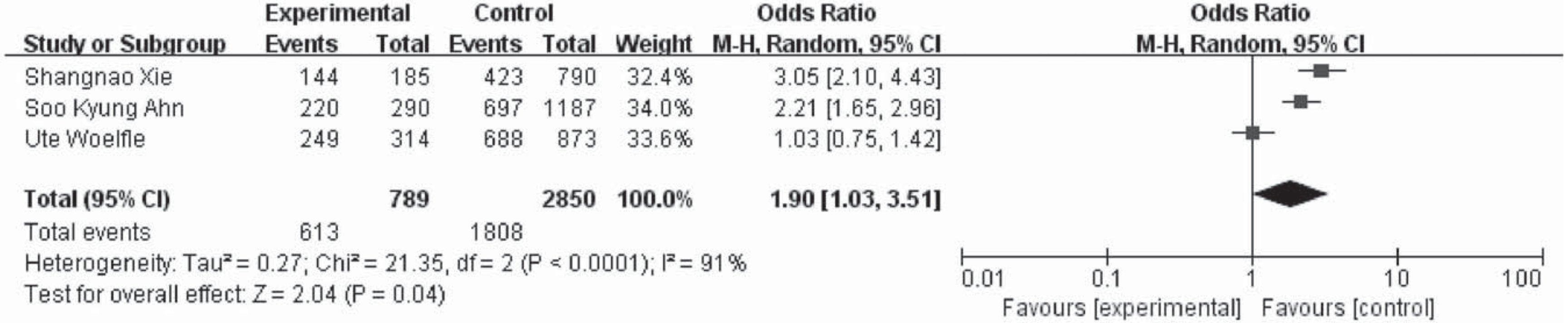

B

Study or Subgroup	Experimental Events	Experimental Total	Control Events	Control Total	Weight	Odds Ratio M-H, Random, 95% CI
Natalia Krawczyk	70	253	12	45	7.2%	1.05 [0.51, 2.15]
Shangnao Xie	137	582	86	393	39.0%	1.10 [0.81, 1.49]
Soo Kyung Ahn	122	577	168	893	53.8%	1.16 [0.89, 1.50]
**Total (95% CI)**		**1412**		**1331**	**100.0%**	**1.13 [0.93, 1.36]**
Total events	329		266			

Heterogeneity: Tau² = 0.00; Chi² = 0.10, df = 2 (P = 0.95); I² = 0%
Test for overall effect: Z = 1.22 (P = 0.22)

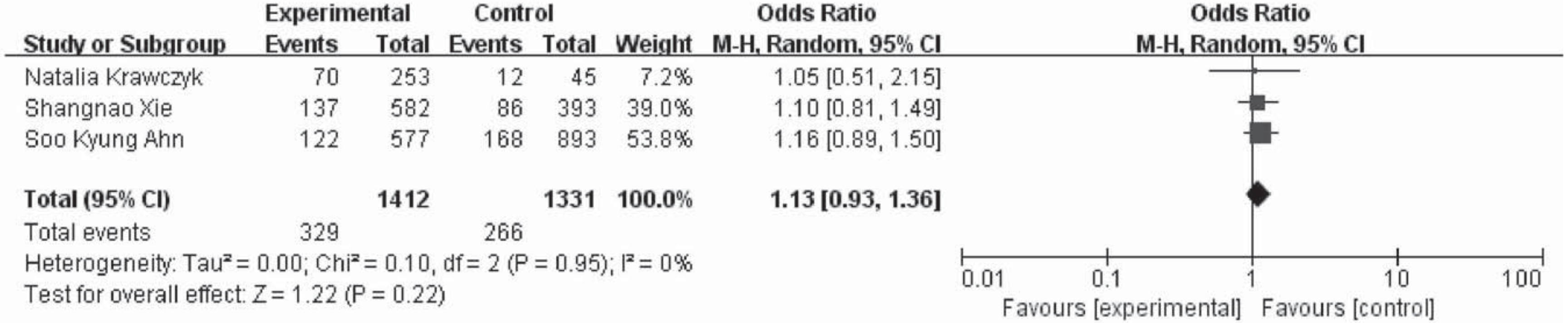

C

Study or Subgroup	Experimental Events	Experimental Total	Control Events	Control Total	Weight	Odds Ratio M-H, Fixed, 95% CI
B.K. Linderholm	40	73	164	336	12.2%	1.27 [0.76, 2.11]
Natalia Krawczyk	11	53	71	245	9.2%	0.64 [0.31, 1.32]
Shangnao Xie	117	475	99	500	33.5%	1.32 [0.98, 1.79]
Soo Kyung Ahn	149	666	137	784	45.0%	1.36 [1.05, 1.76]
**Total (95% CI)**		**1267**		**1865**	**100.0%**	**1.27 [1.07, 1.52]**
Total events	317		471			

Heterogeneity: Chi² = 3.81, df = 3 (P = 0.28); I² = 21%
Test for overall effect: Z = 2.66 (P = 0.008)

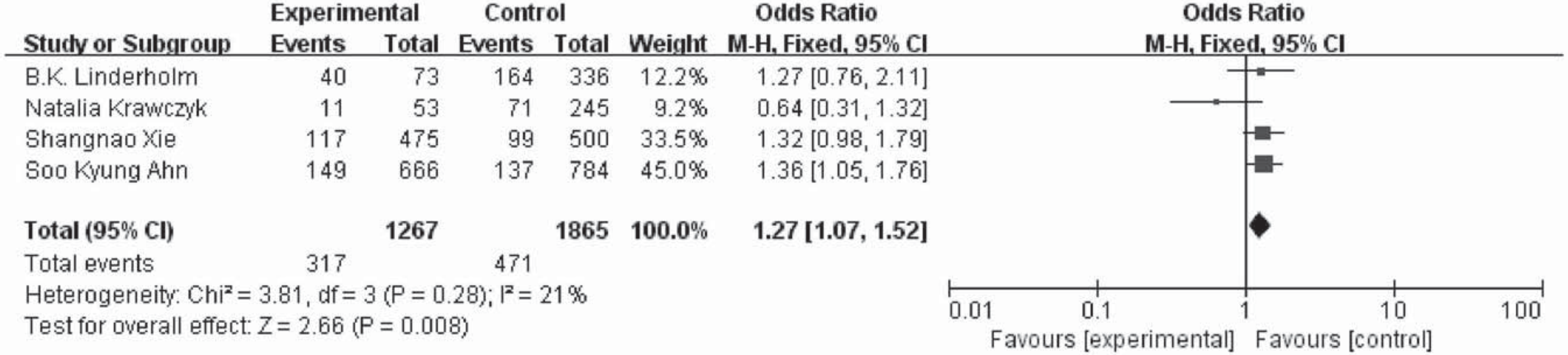

D

Study or Subgroup	Experimental Events	Experimental Total	Control Events	Control Total	Weight	Odds Ratio M-H, Fixed, 95% CI
Natalia Krawczyk	12	52	69	232	17.5%	0.71 [0.35, 1.43]
Shangnao Xie	39	140	184	835	34.3%	1.37 [0.91, 2.05]
Soo Kyung Ahn	56	225	232	1242	48.2%	1.44 [1.03, 2.02]
**Total (95% CI)**		**417**		**2309**	**100.0%**	**1.29 [1.01, 1.64]**
Total events	107		485			

Heterogeneity: Chi² = 3.29, df = 2 (P = 0.19); I² = 39%
Test for overall effect: Z = 2.05 (P = 0.04)

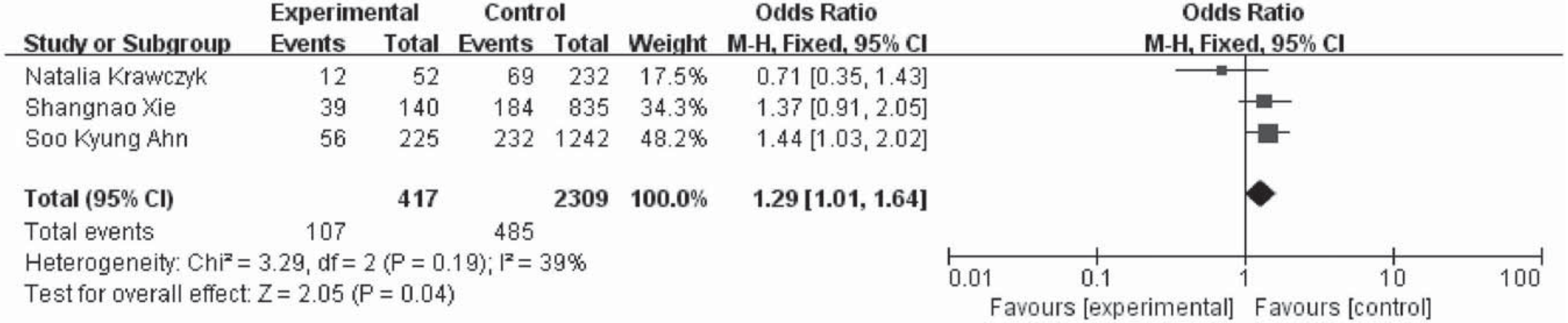

A

Study or Subgroup	Experimental Events	Experimental Total	Control Events	Control Total	Weight	Odds Ratio M-H, Random, 95% CI
B.K. Linderholm	172	336	33	73	19.6%	1.27 [0.76, 2.11]
Gerhard Schaller	113	134	21	134	18.5%	28.95 [14.99, 55.95]
Shangnao Xie	162	220	393	755	20.6%	2.57 [1.85, 3.59]
Soo Kyung Ahn	935	1124	197	269	20.7%	1.81 [1.32, 2.47]
Ute Woelfle	877	1083	206	269	20.7%	1.30 [0.95, 1.79]
**Total (95% CI)**		**2897**		**1500**	**100.0%**	**2.83 [1.32, 6.06]**
Total events	2259		850			

Heterogeneity: Tau² = 0.70; Chi² = 76.09, df = 4 (P < 0.00001); I² = 95%
Test for overall effect: Z = 2.68 (P = 0.007)

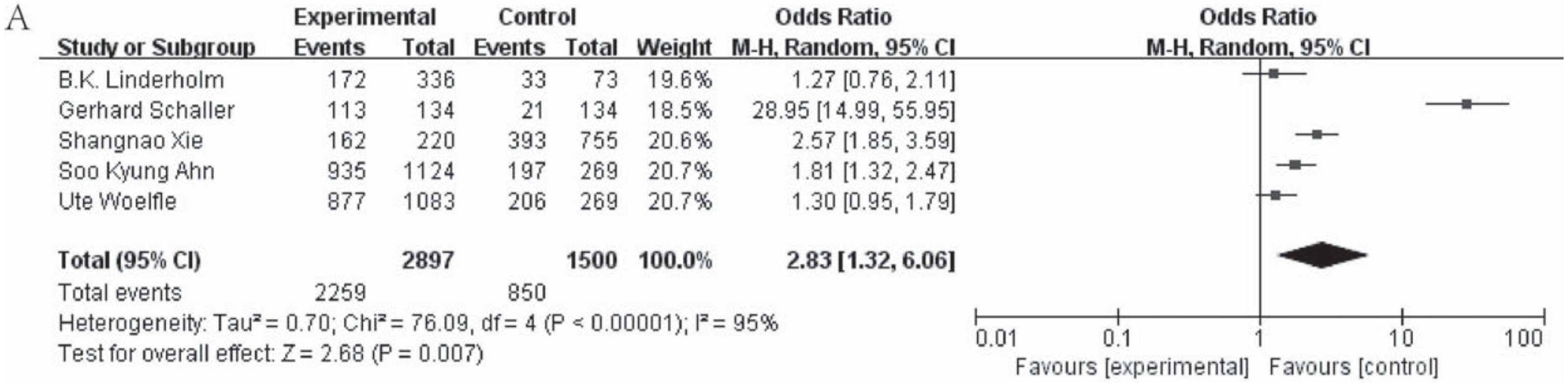

B

Study or Subgroup	Experimental Events	Experimental Total	Control Events	Control Total	Weight	Odds Ratio M-H, Random, 95% CI
B.K. Linderholm	58	80	22	80	14.2%	6.95 [3.47, 13.91]
Gerhard Schaller	28	84	54	214	15.8%	1.48 [0.86, 2.56]
Natalia Krawczyk	93	169	104	219	17.4%	1.35 [0.90, 2.02]
Shangnao Xie	94	197	362	768	18.2%	1.02 [0.75, 1.40]
Soo Kyung Ahn	156	214	429	891	18.0%	2.90 [2.08, 4.02]
Ute Woelfle	83	134	51	134	16.4%	2.65 [1.62, 4.34]
**Total (95% CI)**		**878**		**2306**	**100.0%**	**2.11 [1.28, 3.46]**
Total events	512		1022			

Heterogeneity: Tau² = 0.33; Chi² = 39.81, df = 5 (P < 0.00001); I² = 87%
Test for overall effect: Z = 2.94 (P = 0.003)

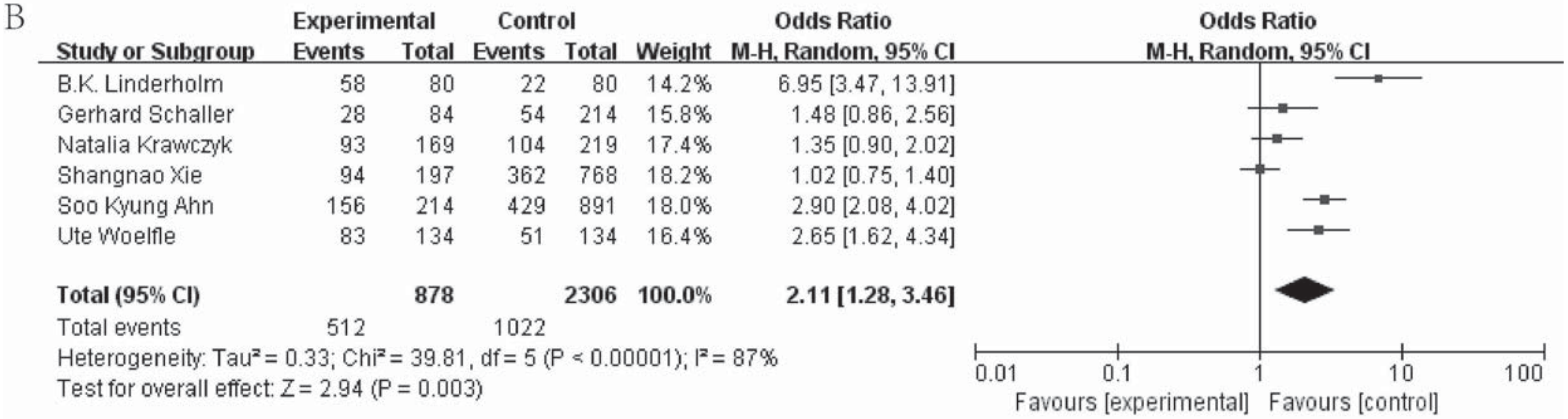

C

Study or Subgroup	Experimental Events	Experimental Total	Control Events	Control Total	Weight	Odds Ratio M-H, Fixed, 95% CI
Natalia Krawczyk	8	30	74	268	9.0%	0.95 [0.41, 2.24]
Shangnao Xie	88	183	315	792	50.4%	1.40 [1.02, 1.94]
Ute Woelfle	284	1022	46	348	40.7%	2.53 [1.80, 3.55]
**Total (95% CI)**		**1235**		**1408**	**100.0%**	**1.82 [1.46, 2.27]**
Total events	380		435			

Heterogeneity: Chi² = 8.30, df = 2 (P = 0.02); I² = 76%
Test for overall effect: Z = 5.33 (P < 0.00001)

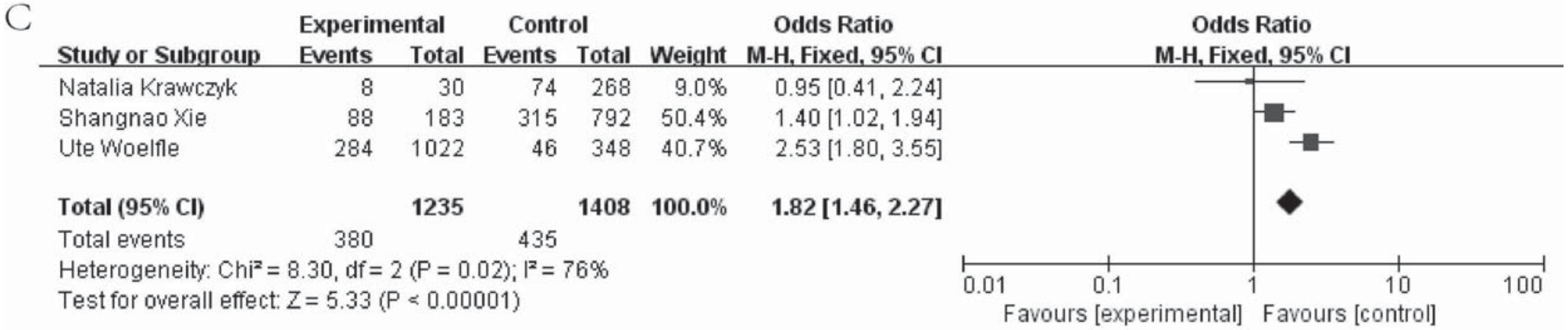

D

Study or Subgroup	Experimental Events	Experimental Total	Control Events	Control Total	Weight	Odds Ratio M-H, Fixed, 95% CI
B.K. Linderholm	82	210	97	205	18.5%	0.71 [0.48, 1.05]
Natalia Krawczyk	29	108	12	63	3.4%	1.56 [0.73, 3.33]
Shangnao Xie	126	221	352	754	21.2%	1.51 [1.12, 2.05]
Soo Kyung Ahn	155	270	520	1127	26.5%	1.57 [1.20, 2.06]
Ute Woelfle	266	369	691	1085	30.3%	1.47 [1.14, 1.91]
**Total (95% CI)**		**1178**		**3234**	**100.0%**	**1.37 [1.19, 1.58]**
Total events	658		1672			

Heterogeneity: Chi² = 12.62, df = 4 (P = 0.01); I² = 68%
Test for overall effect: Z = 4.31 (P < 0.0001)

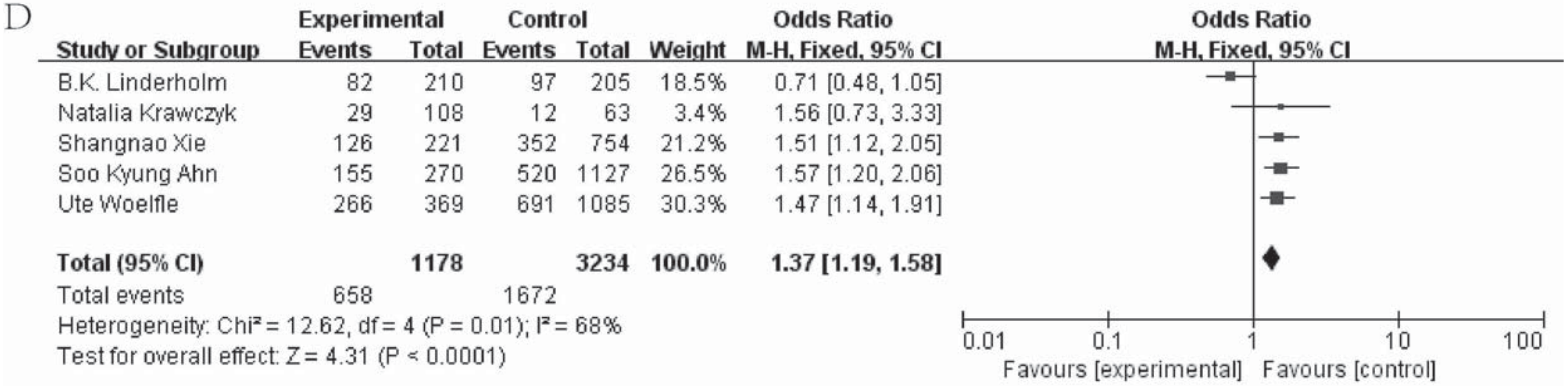

A

Study or Subgroup	log[Hazard Ratio]	SE	Weight	Hazard Ratio IV, Random, 95% CI
**1.3.1 Asian**				
Maria Hagg Olofssion	-0.6777807	0.44985408	5.6%	0.51 [0.21, 1.23]
MASAHIROTAKADA	-0.51768956	0.23010115	11.6%	0.60 [0.38, 0.94]
Shangnao Xie	0.21563756	0.09250275	17.0%	1.24 [1.03, 1.49]
Soo Kyung Ahn	0.19672872	0.08849245	17.1%	1.22 [1.02, 1.45]
**Subtotal (95% CI)**			**51.2%**	**0.97 [0.72, 1.32]**
Heterogeneity: Tau² = 0.06; Chi² = 12.55, df = 3 (P = 0.006); I² = 76%				
Test for overall effect: Z = 0.18 (P = 0.85)				
**1.3.2 Caucasian**				
Faruk Tas	0.17897695	0.14407421	15.0%	1.20 [0.90, 1.59]
Gerhard Schaller	-0.7597496	0.62566207	3.4%	0.47 [0.14, 1.59]
Natalia Krawczyk	0.5289167	0.1856484	13.3%	1.70 [1.18, 2.44]
Ute Woelfle	-0.30103	0.09207297	17.0%	0.74 [0.62, 0.89]
**Subtotal (95% CI)**			**48.8%**	**1.03 [0.65, 1.63]**
Heterogeneity: Tau² = 0.16; Chi² = 20.90, df = 3 (P = 0.0001); I² = 86%				
Test for overall effect: Z = 0.13 (P = 0.90)				
**Total (95% CI)**			**100.0%**	**0.99 [0.77, 1.27]**
Heterogeneity: Tau² = 0.09; Chi² = 38.33, df = 7 (P < 0.00001); I² = 82%				
Test for overall effect: Z = 0.05 (P = 0.96)				
Test for subgroup differences: Chi² = 0.04, df = 1 (P = 0.84), I² = 0%				

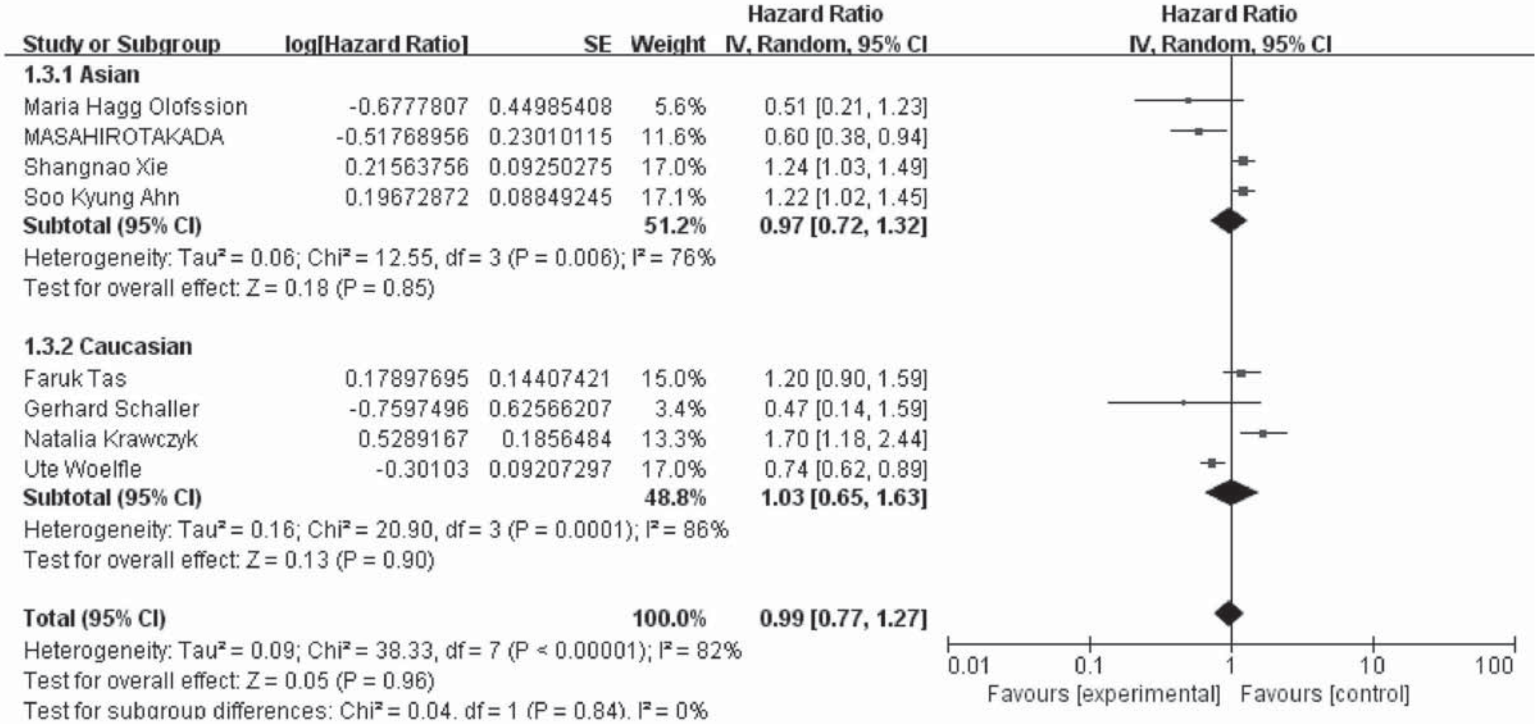

B

Study or Subgroup	log[Hazard Ratio]	SE	Weight	Hazard Ratio IV, Random, 95% CI
**1.6.1 >60 months**				
Gerhard Schaller	-0.7597496	0.62566207	3.4%	0.47 [0.14, 1.59]
Maria Hagg Olofssion	-0.6777807	0.44985408	5.6%	0.51 [0.21, 1.23]
Shangnao Xie	0.21563756	0.09250275	17.0%	1.24 [1.03, 1.49]
Soo Kyung Ahn	0.19672872	0.08849245	17.1%	1.22 [1.02, 1.45]
**Subtotal (95% CI)**			**43.0%**	**1.12 [0.89, 1.42]**
Heterogeneity: Tau² = 0.02; Chi² = 6.08, df = 3 (P = 0.11); I² = 51%				
Test for overall effect: Z = 0.99 (P = 0.32)				
**1.6.2 <60 months**				
Faruk Tas	0.17897695	0.14407421	15.0%	1.20 [0.90, 1.59]
MASAHIROTAKADA	-0.51768956	0.23010115	11.6%	0.60 [0.38, 0.94]
Natalia Krawczyk	0.5289167	0.1856484	13.3%	1.70 [1.18, 2.44]
Ute Woelfle	-0.30103	0.09207297	17.0%	0.74 [0.62, 0.89]
**Subtotal (95% CI)**			**57.0%**	**0.98 [0.64, 1.48]**
Heterogeneity: Tau² = 0.15; Chi² = 23.29, df = 3 (P < 0.0001); I² = 87%				
Test for overall effect: Z = 0.11 (P = 0.91)				
**Total (95% CI)**			**100.0%**	**0.99 [0.77, 1.27]**
Heterogeneity: Tau² = 0.09; Chi² = 38.33, df = 7 (P < 0.00001); I² = 82%				
Test for overall effect: Z = 0.05 (P = 0.96)				
Test for subgroup differences: Chi² = 0.34, df = 1 (P = 0.56), I² = 0%				

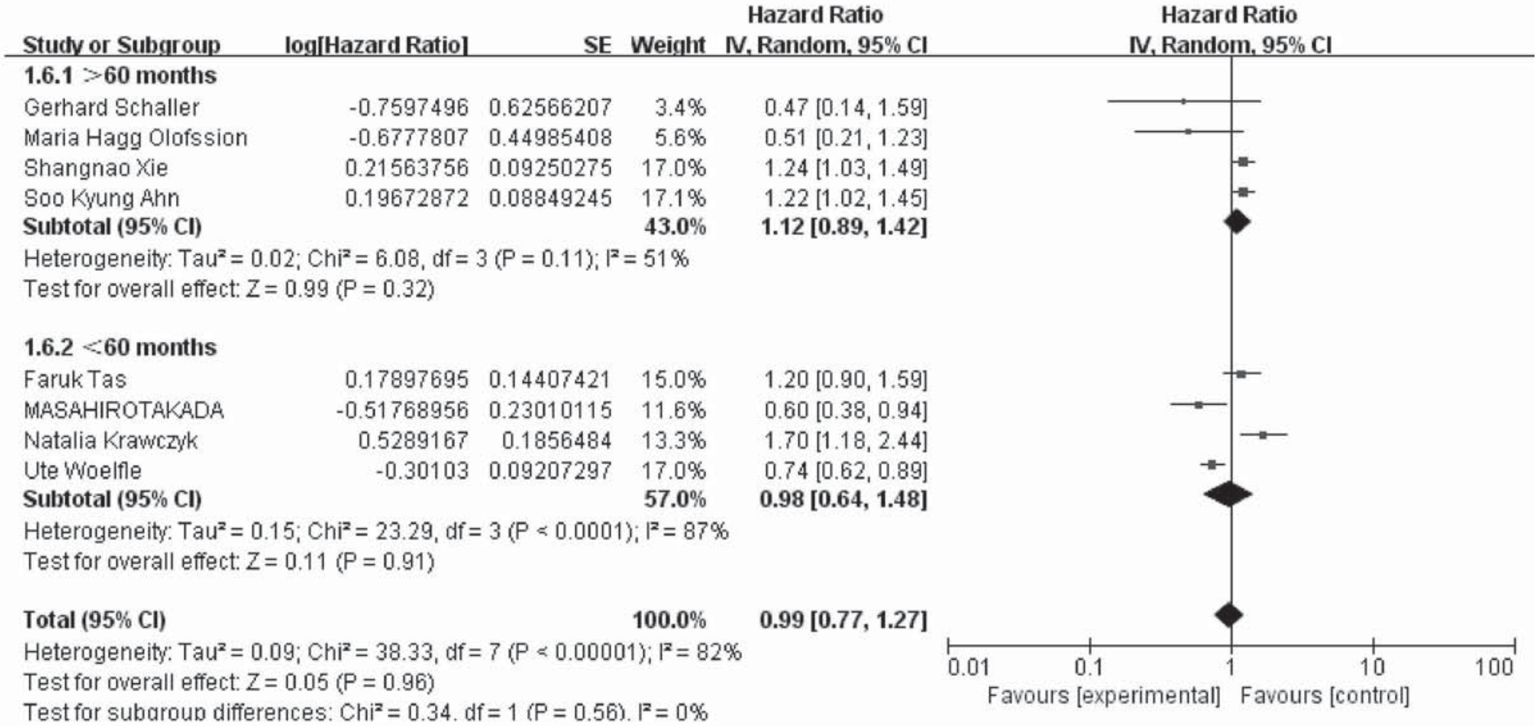

C

Study or Subgroup	log[Hazard Ratio]	SE	Weight	Hazard Ratio IV, Random, 95% CI
**1.5.1 >50**				
Faruk Tas	0.17897695	0.14407421	15.0%	1.20 [0.90, 1.59]
Gerhard Schaller	-0.7597496	0.62566207	3.4%	0.47 [0.14, 1.59]
MASAHIROTAKADA	-0.51768956	0.23010115	11.6%	0.60 [0.38, 0.94]
Natalia Krawczyk	0.5289167	0.1856484	13.3%	1.70 [1.18, 2.44]
Ute Woelfle	-0.30103	0.09207297	17.0%	0.74 [0.62, 0.89]
**Subtotal (95% CI)**			**60.3%**	**0.92 [0.62, 1.37]**
Heterogeneity: Tau² = 0.15; Chi² = 24.39, df = 4 (P < 0.0001); I² = 84%				
Test for overall effect: Z = 0.39 (P = 0.69)				
**1.5.2 <50**				
Maria Hagg Olofssion	-0.6777807	0.44985408	5.6%	0.51 [0.21, 1.23]
Shangnao Xie	0.21563756	0.09250275	17.0%	1.24 [1.03, 1.49]
Soo Kyung Ahn	0.19672872	0.08849245	17.1%	1.22 [1.02, 1.45]
**Subtotal (95% CI)**			**39.7%**	**1.18 [0.96, 1.44]**
Heterogeneity: Tau² = 0.01; Chi² = 3.80, df = 2 (P = 0.15); I² = 47%				
Test for overall effect: Z = 1.59 (P = 0.11)				
**Total (95% CI)**			**100.0%**	**0.99 [0.77, 1.27]**
Heterogeneity: Tau² = 0.09; Chi² = 38.33, df = 7 (P < 0.00001); I² = 82%				
Test for overall effect: Z = 0.05 (P = 0.96)				
Test for subgroup differences: Chi² = 1.14, df = 1 (P = 0.29), I² = 12.4%				

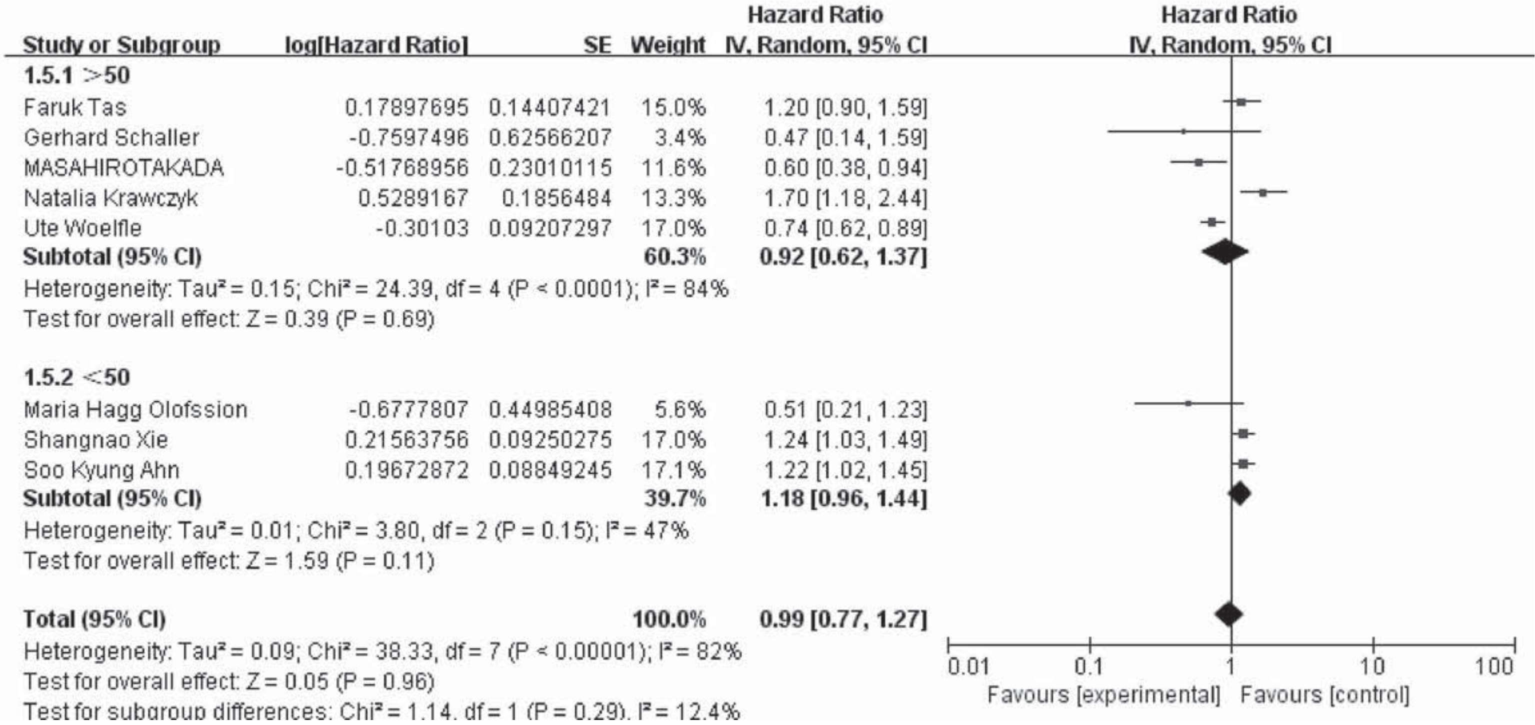

```
Begg's Test

  adj. Kendall's Score (P-Q) =       -2
          Std. Dev. of Score =     8.08
           Number of Studies =        8
                          z  =    -0.25
                   Pr > |z|  =    0.805
                          z  =     0.12 (continuity corrected)
                   Pr > |z|  =    0.902 (continuity corrected)
```

Egger's test

Std_Eff	Coef.	Std. Err.	t	P>|t|	[95% Conf.	Interval]
slope	.193127	.233085	0.83	0.439	-.3772115	.7634655
bias	-1.240069	1.779134	-0.70	0.512	-5.593453	3.113315

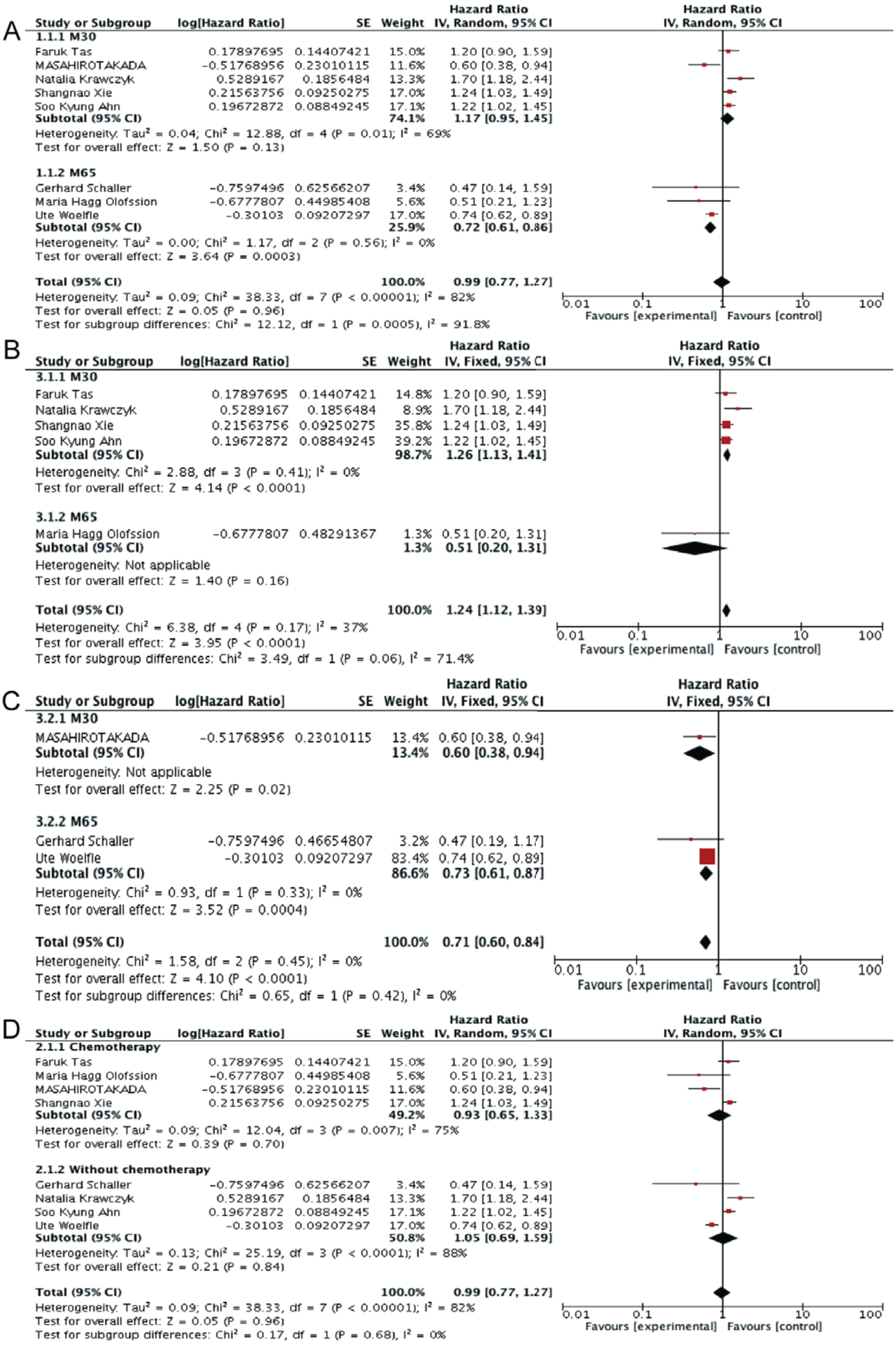
A
Study or Subgroup log[Hazard Ratio] SE Weight Hazard Ratio IV, Random, 95% CI
Hazard Ratio IV, Random, 95% CI
1.1.1 M30
Faruk Tas 0.17897695 0.14407421 15.0% 1.20 [0.90, 1.59]
MASAHIROTAKADA -0.51768956 0.23010115 11.6% 0.60 [0.38, 0.94]
Natalia Krawczyk 0.5289167 0.1856484 13.3% 1.70 [1.18, 2.44]
Shangnao Xie 0.21563756 0.09250275 17.0% 1.24 [1.03, 1.49]
Soo Kyung Ahn 0.19672872 0.08849245 17.1% 1.22 [1.02, 1.45]
Subtotal (95% CI) 74.1% 1.17 [0.95, 1.45]
Heterogeneity: Tau² = 0.04; Chi² = 12.88, df = 4 (P = 0.01); I² = 69%
Test for overall effect: Z = 1.50 (P = 0.13)
1.1.2 M65
Gerhard Schaller -0.7597496 0.62566207 3.4% 0.47 [0.14, 1.59]
Maria Hagg Olofssion -0.6777807 0.44985408 5.6% 0.51 [0.21, 1.23]
Ute Woelfle -0.30103 0.09207297 17.0% 0.74 [0.62, 0.89]
Subtotal (95% CI) 25.9% 0.72 [0.61, 0.86]
Heterogeneity: Tau² = 0.00; Chi² = 1.17, df = 2 (P = 0.56); I² = 0%
Test for overall effect: Z = 3.64 (P = 0.0003)
Total (95% CI) 100.0% 0.99 [0.77, 1.27]
Heterogeneity: Tau² = 0.09; Chi² = 38.33, df = 7 (P < 0.00001); I² = 82%
Test for overall effect: Z = 0.05 (P = 0.96)
Test for subgroup differences: Chi² = 12.12, df = 1 (P = 0.0005), I² = 91.8%
0.01 0.1 1 10 100
Favours [experimental] Favours [control]
B
Study or Subgroup log[Hazard Ratio] SE Weight Hazard Ratio IV, Fixed, 95% CI
Hazard Ratio IV, Fixed, 95% CI
3.1.1 M30
Faruk Tas 0.17897695 0.14407421 14.8% 1.20 [0.90, 1.59]
Natalia Krawczyk 0.5289167 0.1856484 8.9% 1.70 [1.18, 2.44]
Shangnao Xie 0.21563756 0.09250275 35.8% 1.24 [1.03, 1.49]
Soo Kyung Ahn 0.19672872 0.08849245 39.2% 1.22 [1.02, 1.45]
Subtotal (95% CI) 98.7% 1.26 [1.13, 1.41]
Heterogeneity: Chi² = 2.88, df = 3 (P = 0.41); I² = 0%
Test for overall effect: Z = 4.14 (P < 0.0001)
3.1.2 M65
Maria Hagg Olofssion -0.6777807 0.48291367 1.3% 0.51 [0.20, 1.31]
Subtotal (95% CI) 1.3% 0.51 [0.20, 1.31]
Heterogeneity: Not applicable
Test for overall effect: Z = 1.40 (P = 0.16)
Total (95% CI) 100.0% 1.24 [1.12, 1.39]
Heterogeneity: Chi² = 6.38, df = 4 (P = 0.17); I² = 37%
Test for overall effect: Z = 3.95 (P < 0.0001)
Test for subgroup differences: Chi² = 3.49, df = 1 (P = 0.06), I² = 71.4%
0.01 0.1 1 10 100
Favours [experimental] Favours [control]
C
Study or Subgroup log[Hazard Ratio] SE Weight Hazard Ratio IV, Fixed, 95% CI
Hazard Ratio IV, Fixed, 95% CI
3.2.1 M30
MASAHIROTAKADA -0.51768956 0.23010115 13.4% 0.60 [0.38, 0.94]
Subtotal (95% CI) 13.4% 0.60 [0.38, 0.94]
Heterogeneity: Not applicable
Test for overall effect: Z = 2.25 (P = 0.02)
3.2.2 M65
Gerhard Schaller -0.7597496 0.46654807 3.2% 0.47 [0.19, 1.17]
Ute Woelfle -0.30103 0.09207297 83.4% 0.74 [0.62, 0.89]
Subtotal (95% CI) 86.6% 0.73 [0.61, 0.87]
Heterogeneity: Chi² = 0.93, df = 1 (P = 0.33); I² = 0%
Test for overall effect: Z = 3.52 (P = 0.0004)
Total (95% CI) 100.0% 0.71 [0.60, 0.84]
Heterogeneity: Chi² = 1.58, df = 2 (P = 0.45); I² = 0%
Test for overall effect: Z = 4.10 (P < 0.0001)
Test for subgroup differences: Chi² = 0.65, df = 1 (P = 0.42), I² = 0%
0.01 0.1 1 10 100
Favours [experimental] Favours [control]
D
Study or Subgroup log[Hazard Ratio] SE Weight Hazard Ratio IV, Random, 95% CI
Hazard Ratio IV, Random, 95% CI
2.1.1 Chemotherapy
Faruk Tas 0.17897695 0.14407421 15.0% 1.20 [0.90, 1.59]
Maria Hagg Olofssion -0.6777807 0.44985408 5.6% 0.51 [0.21, 1.23]
MASAHIROTAKADA -0.51768956 0.23010115 11.6% 0.60 [0.38, 0.94]
Shangnao Xie 0.21563756 0.09250275 17.0% 1.24 [1.03, 1.49]
Subtotal (95% CI) 49.2% 0.93 [0.65, 1.33]
Heterogeneity: Tau² = 0.09; Chi² = 12.04, df = 3 (P = 0.007); I² = 75%
Test for overall effect: Z = 0.39 (P = 0.70)
2.1.2 Without chemotherapy
Gerhard Schaller -0.7597496 0.62566207 3.4% 0.47 [0.14, 1.59]
Natalia Krawczyk 0.5289167 0.1856484 13.3% 1.70 [1.18, 2.44]
Soo Kyung Ahn 0.19672872 0.08849245 17.1% 1.22 [1.02, 1.45]
Ute Woelfle -0.30103 0.09207297 17.0% 0.74 [0.62, 0.89]
Subtotal (95% CI) 50.8% 1.05 [0.69, 1.59]
Heterogeneity: Tau² = 0.13; Chi² = 25.19, df = 3 (P < 0.0001); I² = 88%
Test for overall effect: Z = 0.21 (P = 0.84)
Total (95% CI) 100.0% 0.99 [0.77, 1.27]
Heterogeneity: Tau² = 0.09; Chi² = 38.33, df = 7 (P < 0.00001); I² = 82%
Test for overall effect: Z = 0.05 (P = 0.96)
Test for subgroup differences: Chi² = 0.17, df = 1 (P = 0.68), I² = 0%
0.01 0.1 1 10 100
Favours [experimental] Favours [control]
